# Effects of an In-home Multicomponent Exergame Training on Physical Functions, Cognition, and Brain Volume of Older Adults: A Randomized Controlled Trial

**DOI:** 10.3389/fmed.2019.00321

**Published:** 2020-01-28

**Authors:** Manuela Adcock, Mélanie Fankhauser, Jennifer Post, Kai Lutz, Leopold Zizlsperger, Andreas R. Luft, Vânia Guimarães, Alexandra Schättin, Eling D. de Bruin

**Affiliations:** ^1^Institute of Human Movement Sciences and Sport, Department of Health Sciences and Technology, ETH Zurich, Zurich, Switzerland; ^2^Cereneo, Center for Neurology and Rehabilitation, Vitznau, Switzerland; ^3^Department of Neurology, University Hospital of Zurich, Zurich, Switzerland; ^4^Fraunhofer Portugal AICOS, Porto, Portugal; ^5^Division of Physiotherapy, Department of Neurobiology, Care Sciences and Society, Karolinska Institutet, Stockholm, Sweden

**Keywords:** physical-cognitive training, exergame, physical functions, cognition, brain volume, healthy aging

## Abstract

Aging is associated with a decline in physical functions, cognition and brain structure. Considering that human life is based on an inseparable physical-cognitive interplay, combined physical-cognitive training through exergames is a promising approach to counteract age-related impairments. The aim of this study was to assess the effects of an in-home multicomponent exergame training on [i] physical and cognitive functions and [ii] brain volume of older adults compared to a usual care control group. Thirty-seven healthy and independently living older adults aged 65 years and older were randomly assigned to an intervention (exergame training) or a control (usual care) group. Over 16 weeks, the participants of the intervention group absolved three home-based exergame sessions per week (à 30–40 min) including Tai Chi-inspired exercises, dancing and step-based cognitive games. The control participants continued with their normal daily living. Pre- and post-measurements included assessments of physical (gait parameters, functional muscle strength, balance, aerobic endurance) and cognitive (processing speed, short-term attention span, working memory, inhibition, mental flexibility) functions. T1-weighted magnetic resonance imaging was conducted to assess brain volume. Thirty-one participants (mean age = 73.9 ± 6.4 years, range = 65–90 years, 16 female) completed the study. Inhibition and working memory significantly improved post-intervention in favor of the intervention group [inhibition: *F*_(1)_ = 2.537, *p* = 0.046, ${\text{n}}_{\text{p}}^{2}$ = 0.11, working memory: *F*_(1)_ = 5.872, *p* = 0.015, ${\text{n}}_{\text{p}}^{2}$ = 0.02]. Two measures of short-term attentional span showed improvements after training in favor of the control group [F_(1)_ = 4.309, *p* = 0.038, ${\text{n}}_{\text{p}}^{2}$ = 0.03, *F*_(1)_ = 8.504, *p* = 0.004, ${\text{n}}_{\text{p}}^{2}$ = 0.04]. No significant training effects were evident for physical functions or brain volume. Both groups exhibited a significant decrease in gray matter volume of frontal areas and the hippocampus over time. The findings indicate a positive influence of exergame training on executive functioning. No improvements in physical functions or brain volume were evident in this study. Better adapted individualized training challenge and a longer training period are suggested. Further studies are needed that assess training-related structural brain plasticity and its effect on performance, daily life functioning and healthy aging.

## Introduction

The worldwide population of older adults is growing fast and concurrently, life expectancy is prolonged leading to an increase of age-related impairments and diseases (1). These degenerative changes affect physical and cognitive functions resulting in impaired daily life functioning including mobility impairments which, in turn, reduce independence and psychological well-being as well as increase health care costs (2). Moreover, aging is accompanied by alterations in brain structure. Significant gray matter (GM) volume reduction in frontal, temporal, and subcortical areas are described in elderly with mild cognitive impairment or dementia as well as in healthy older adults (3). The diminished brain structures seem to be related to behavioral impairments and might translate to cognitive and/or physical dysfunctioning (4). However, a considerable inter-individual variation exists in age-related declines (5). Thus, other factors than age itself seem to influence functionality in older age, for example lifestyle and behavior (6). Human brain and body are plastic and adapt in response to experiences and stimulation (7). Also in higher age, experience-based plasticity can be observed on behavioral and neuronal level (8). Nevertheless, a knowledge gap exists regarding the optimal ways for influencing neuronal plasticity. To conclude, a strong need exists to support healthy aging by identifying effective strategies, which trigger experience-based (neuro)plasticity.

One behavioral strategy to promote healthy aging is applying physical exercise. Many studies have shown that physical activity has various positive effects on the human body (9). Physical exercise improves the cardiovascular and respiratory system, the metabolism, the immune system and body composition (10). Moreover, cross-sectional as well as longitudinal and intervention studies revealed positive effects of physical activity and exercising on psychological well-being and cognition of older adults (11, 12). On brain level, animal and human studies demonstrated positive effects on various brain structures, particularly a brain volume increase in the frontal cortex and the hippocampus, areas that are most affected during the aging process (13–15). Although the underlying mechanisms of these brain alterations are not yet fully understood and difficult to explore *in vivo* in humans, physical exercise is known to induce a cascade of cellular and molecular processes that support brain plasticity (16, 17). Changes in neurotransmitters and/or neurotrophic factors are supposed to play a critical role in training-related neuroplasticity (18, 19). For example, exercising increases the expression of neurotrophins such as vascular endothelial growth factor (VEGF), brain-derived neurotrophic factor (BDNF) or insulin-like growth factor 1 (IGF-1), which might trigger neuroplasticity (20). Synaptic changes e.g., strengthening of existing connections or building new synapses (synaptogenesis), the creation of new blood vessels (angiogenesis) and the development of new neurons (neurogenesis) and glia cells (gliagenesis) are discussed as underlying mechanisms for neuroplasticity (17, 21–25).

Besides physical exercise, challenging environments and cognitive training are also discussed to be able to enhance cognitive performance and positively change certain brain structures. The results of reviews and meta-analyses show that older adults can benefit from cognitive training (26, 27). Cognitive training seems to be effective in improving various aspects of cognitive functioning including memory, executive functions, attention, visuo-spatial skills and processing speed (28). Regarding neuronal measures, a positive influence on gray and white brain matter has been reported after cognitive training in older adults, mainly in frontal, temporal (including hippocampus), and parietal areas (29–33). Animal studies, with mice or rats raised in enriched environments (environments with various objects as toys, tunnels, and mirrors providing cognitive stimulation), showed altered brain structure and better cognitive performance (34–36). As underlying mechanism, neurogenesis, synaptogenesis and adaptations in astrocytes are discussed (34–37). Moreover, BDNF production has been shown to be increased by cognitive stimulation (37, 38).

Considering the significant impact of physical activity and cognitive training on several health aspects, it has been assumed that a combination of physical exercise with cognitive stimulation poses a promising training approach with superior benefits due to several reasons (39–44). First, most daily life activities require the simultaneous performance of cognitive and physical functions (45). Thus, combined physical-cognitive training has ecological validity and is close to daily life requirements. This might enhance transfer effects and perceived meaningfulness of training (43). Second, the inseparable physical-cognitive interplay of human life can be described also from a more biological perspective: Muscles and movements are controlled by the central nervous system, whereas feedback from peripheral structures as muscles and sensory organs influence brain activity (19). Combined training targets this interplay. Third, synergistic effects of combined training are assumed: Physical activity seems to trigger neuroplastic effects, but cognitive stimulation might be crucial to guide and consolidate these effects (46, 47).

A promising approach for interactive physical-cognitive training are so-called exergames. Exergames are digital games, which require bodily movements to play the game (48). In the virtual reality/game scenario, cognitive stimulation may be, more or less explicitly and specifically, embedded. Exergames are known to increase training motivation due to “gamification” of training leading to engaging and fun gameplay (49, 50). Depending on the applied technology, exergame training might be conducted in an in-home setting that overcomes accessibility barriers (51). In general, exergame training has been shown to improve both, cognitive functions (mainly executive functions) (52, 53) and physical functions (cardiovascular or musculoskeletal system) depending on the physical components and exercises that are integrated in the training (e.g., stepping or cycling) (54–56). Compared to traditional cognitive or physical training (e.g., aerobic training), exergaming seems to be equally or slightly more effective for cognitive enhancement in older adults (52, 53). On brain level, two studies assessed changes in brain structure after exergame training in older adults (57, 58). To our knowledge, however, no past research has investigated the effect of home-based exergame training, tailored to older adults' needs, on physical and cognitive functions and on brain structure of the elderly.

Considering the theoretical background from human movement science and neuropsychology together with the art of game design and the needs of older adults, the Active@Home exergame was developed. This exergame enables multicomponent training including strength, balance and cognitive training. Usability and feasibility of the newly developed exergame was tested previously (59). Results showed that the Active@Home exergame is a usable and feasible home-based training for older adults. Consequently, the next step in the development process is to conduct a study assessing the effectiveness of the exergame training.

The primary objective of this study was to assess the effects of this in-home multicomponent exergame training on physical and cognitive functions in healthy older adults. The secondary objective included the application of magnetic resonance imaging (MRI) and voxel-based morphometry (VBM) to evaluate the impact of exergame training on brain volume in healthy older adults. We hypothesized that the Active@Home exergame training would enhance physical and cognitive functions as well as positively influence brain volume compared to a passive control group in older adults.

## Materials and Methods

### Participants

Participants were recruited from June to August 2018 through public advertisements in local newspapers, senior organizations, physiotherapy and medical centers as well as supermarkets. All potential participants were screened for eligibility using the Mini Mental State Examination (MMSE) to assess cognitive status and a health questionnaire to assess anthropometric and health data and medical history. Participants fulfilling all of the following inclusion criteria were eligible for the study: (1) age ≥ 65 years, (2) living independently, (3) healthy by self-report, (4) able to stand at least for 10 minutes without assistance, (5) access to a TV with HDMI connection. Participants exhibiting at least one of the following criteria were excluded from the study: (1) cognitive impairments (MMSE ≤ 23 points), (2) mobility impairments that prevent training participation, (3) severe and uncontrolled health problems (e.g., recent cardiac infarction, uncontrolled diabetes or hypertension), (4) orthopedic disease that prevents training participation, (5) neurological disease (e.g., history of stroke or epilepsy, Parkinson's disease, Alzheimer disease or other forms of dementia), (6) acute severe, rapidly progressive or terminal illness, (7) intake of any psychoactive substances (e.g., neuroleptics, antidepressants), (8) active participation in a supervised physical or cognitive training. Consumption of nicotine or caffeine was not defined as an exclusion criterion, but participants were asked to consume the usual amount before measurements to avoid deprivations states. Regarding MRI measurements, participants with claustrophobia or magnetic metal pieces in the body were excluded.

Written informed consent was obtained from all participants before any measurements. The intended study sample size of 40 participants was determined by the number of available training/exergame systems. Participants received no payment or refund for traveling expenses but a detailed feedback on personal performance and general study outcomes at the end of the trial.

### Study Design and Procedure

This randomized controlled trial (RCT) was a clinical phase III study (60) and used an experimental mixed design with time (pre, post) as within-subject factor and group (training, control) as between-subject factor.

The eligible participants were randomly allocated to either the intervention or the control group using a computer-based randomization tool (www.randomization.com). To counteract size imbalances of the groups, permutated block randomization method with block size of four was used (61). The allocation sequence was kept by an independent study investigator whereas participant enrollment and assignment were done by other study investigators. Blinding of investigators was not possible since they were responsible for both, conduction of measurements and supervision during the intervention period. Blinding of participants was not possible since a passive control group was used.

Assessments were performed at baseline (pre) and at the end (post) of the intervention phase within a timeframe of 2 weeks and were conducted at the cereneo (Swiss clinic and research institute at Vitznau, Lucerne, Switzerland). Pre- and post-measurements were conducted at approximately the same daytime for each participant. The intervention period lasted 16–18 weeks (a maximum of 2 weeks holiday interruption was allowed) and took place from July 2018 until December 2018.

The participants of the training group trained three times per week for 30–40 min during 4 months resulting in a total of 48 training sessions. Training frequency and duration followed previous exergame studies illustrating positive effects in older adults (40) and current recommendations for physical activity and fall prevention in elderly (62, 63). The training was conducted at participants' home. For installation and instruction, the study investigators visited participants at home. The training sessions were scheduled individually by participants considering a guideline of three training sessions per week and no more than one training session per day. Furthermore, participants received written recommendations how to progressively adapt the training challenge whereas a moderate training intensity was targeted (64). All training sessions were recorded by participants on a training diary. The participants of the control group were instructed to continue with their normal daily living. At study end, Active@Home training systems were provided to the control group members to enable a voluntary 4-month training period.

All participants (training and control group) were called biweekly to monitor for deviations in their usual patterns of physical and cognitive activities (on top of the study-related activity). Intense physical activity (e.g., running or playing tennis) was separated from moderate physical activity (e.g., walking or gardening). Examples for cognitive activities were playing chess, learning a language or using an app for playing games. Furthermore, participants in the training group were asked about potential safety issues during training and were supported in case technical or content related issues arose. A total of eight phone calls per participant were absolved.

All procedures were carried out in accordance with the Declaration of Helsinki and were approved by the ethics committee of Nordwest- und Zentralschweiz (EKNZ, 2018-00510). The study was registered at the Clinical Trial Register (www.clinicaltrials.gov, NCT03676452).

### Training Intervention - Active@Home Exergame

The Active@Home exergame is a technology-based multicomponent in-home training for older adults to train strength, balance and cognition. It consists of the following three components: (1) Tai Chi-inspired exercises, (2) dancing, and (3) step-based cognitive games. Tai Chi-inspired exercises are a combination of lower-limb and core strength exercises with Tai Chi movement elements performed in three different stance positions (squat, plié, and lunge). Dancing exercises are based on dances such as Bachata, Disco Fox, Salsa, Waltz, Chachacha, and Jive and require motor components of balance, coordination, and agility (65). A total of 18 different step-based cognitive games target specific attentional and executive functions; e.g., selective and divided attention, interference control, cognitive flexibility, and working memory. By stepping forward, backward, and to the right or left side, the cognitive games are played and controlled. In the Active@Home exergame, physical and cognitive functions are trained in a combined approach where physical-cognitive interplay is required in all training components. For Tai Chi-inspired and dance exercises, the cognitive demands might be lower than for the step-based specific cognitive games but nevertheless, cognitive functions are required for movement imitation, control and coordination (66, 67). In contrast, the step-based games might be less physically demanding than Tai Chi-inspired and dance exercises, however, movements (steps) are required to play the games. The training content of the Active@Home exergame is based on current recommendations for exercising in older age (63, 68–70).

**Figure 1:**
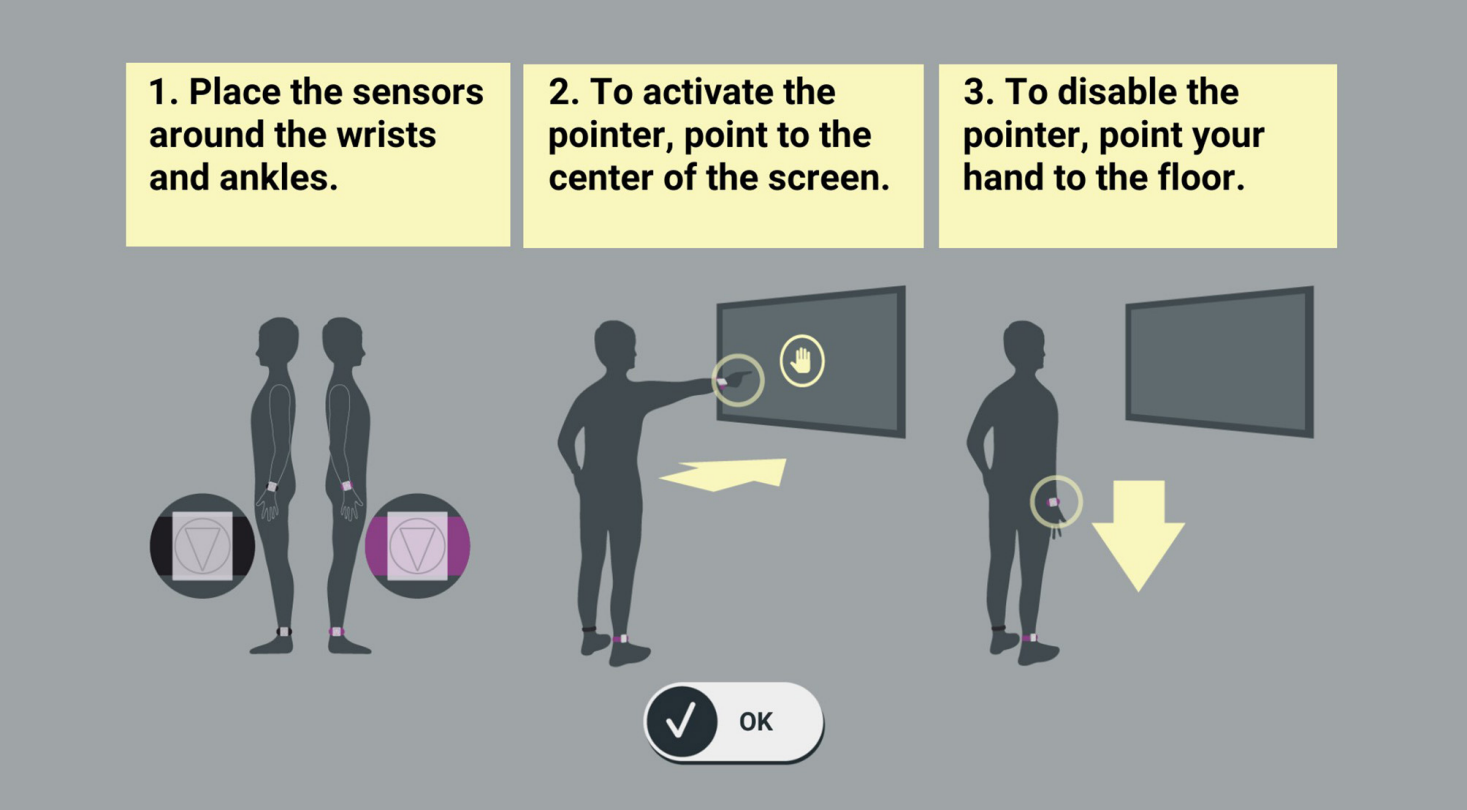
Set up and navigation in the Active@Home exergame. The hardware of the Active@Home exergame consists of four inertial measurement units (IMUs). For movement evaluation, participants wore the sensors at wrists and ankles. Color and size of the bracelet helped to distinguish the position of each IMU. The IMUs were connected via Bluetooth to a HDMI dongle. This dongle was inserted into a television and provided the exergame software. The game interface was presented on the TV screen. By pointing the IMU on the right wrist horizontally to the TV screen, a “hand mouse” got activated for navigation through the game.

The hardware of the Active@Home exergame consists of four inertial measurement units (IMUs) providing both accelerometer and gyroscope assessments. For movement evaluation, participants wore the IMUs at wrists and ankles attached with a silicone slap band. Color and size of the silicon bracelet helped to distinguish the position/placement of each IMU. The IMUs were connected via Bluetooth to a HDMI dongle. This dongle was inserted into a television (TV) and provided the exergame software. The game interface was presented on the TV screen. By pointing the IMU on the right wrist horizontally to the TV screen, a “hand mouse” got activated for navigation through the game (see Figure 1).

The story of the exergame was about traveling in Europe and to train in several different cities (London, Paris, Amsterdam, Rome, Porto, and Zurich). Exercises were instructed by a cartoon-based instructor and were accentuated with background music (71). Moreover, the exergame implemented some basic training principles which are important for effective training such as feedback, optimal challenge and progression, and variety (72). A multimodal feedback system based on symbols, sounds, performances scores and a color code was included. To ensure optimal challenge and progression, several difficulty levels for the exercises were developed. Progression was reached through more complex or faster movements. Each training session included Tai Chi-inspired exercises, dancing and step-based cognitive games to an equal amount. It was recommended to play through all levels of one city before switching to the next city whereas the order of the cities was predefined based on progressive difficulty. Additionally, before changing the level, one level should have been trained at least in two separated sessions. The current difficulty level should always provide an optimal challenge avoiding under- or overload.

### Outcome Measures

#### Assessments of Physical Functions

##### Gait analysis

Parameters of gait kinematics were measured using the wearable Physilog®5 (Gait Up Sàrl, Lausanne, Switzerland), which has been shown to reliably and validly measure gait performance (73). The Physilog®5s were clipped to the top of the right and left shoe (forefoot). Participants were required to walk a 10 m straight and flat distance six times consecutively to achieve a walkway of 60 m resulting in more than 20 gait cycles, ensuring reliable assessment of gait variability (74). Two walking conditions were used: (1) single-task walking (ST): participants were instructed to walk at preferred speed without talking; (2) dual-task walking (DT): participants were instructed to walk at preferred speed and simultaneously count backwards (cognitive task) in steps of seven from a randomly given number between 200 and 250. In this condition, participants were asked to count aloud and perform both tasks concurrently and not to prioritize one task above the other. This is a common method to measure multitasking capabilities.

For further analysis, data were transferred to the computer via USB port. Two walking cycles for initiation and termination and turns were discarded in order to analyse steady state walking. For this study, the focus was on the following parameters: speed [m/s], stride length [m], stride time [s], and minimal toe clearance [cm]. For each gait parameter, mean values of the left and right foot were determined for both walking conditions and coefficients of variation (CV) were calculated for all parameters: CV [%] = (standard deviation)/mean × 100. Dual-task costs (DTC) were calculated as a percentage of loss of the DT relative to the ST performance according to the formula: DTC [%] = (ST—DT)/ST × 100.

##### Short Physical Performance Battery (only balance test)

To assess balance, a subtest of the Short Physical Performance Battery (SPPB) was applied (Interclass Correlation Coefficient of 0.88–0.92) (75, 76). The balance test of the SPPB includes: standing in (1) feet side-by-side position, (2) semi-tandem stance, and (3) full-tandem stance. Each position should be held unsupported for 10 s. The performance was rated with a score ranging from zero (“not able to complete the tasks”) to four points (“good balance function”). In line with previous studies (77), we extended the balance test with two additional tasks to avoid ceiling effects. The first additional task was a 20 s single-leg stance (with preferred leg) where two points were achieved for reaching 20 s, one point for 10–20 s and zero points for <10 s. The second additional task was a single-leg stance (with preferred leg) with eyes closed where one point was assigned for every 5 s of successful task achievement. Three trials were conducted for each additional task whereas the best trial counted. For the extended version of the subtest, the maximum point score is unlimited with higher scores meaning better balance functioning. The total score of the extended subtest was calculated for the analysis.

##### Senior Fitness Test (two subtests)

Two subtests of the Senior Fitness Test (SFT) were used to assess functional lower body strength and aerobic endurance (Interclass Correlation Coefficient of 0.93–0.98) (78, 79). In the 30 s chair rises test, the participants started in a standing position in front of a chair with arms crossed in front of the chest and performed as many full “chair rises” as possible in 30 s. A full chair rise was defined as sitting down on the chair and standing up, ending in an upright position again. The number of completed full chair rises in 30 s was counted.

In the 2 min stepping test, the participants were asked to alternatingly step with both legs in place as many times as possible in 2 min while reaching a predefined individual height with the knees. This threshold height was calculated by means of the height from the floor to the middle of the thigh (midway between the iliac crest and the upper patella). For the analysis, the number of steps with the starting leg was counted during the 2 min whereas a step was valid when the knee was reaching the required height (marked with a tape on the wall).

#### Assessments of Cognitive Functions

##### Victoria Stroop Test

To assess response inhibition and interference control, the Victoria Stroop Test (VST) was used, a tool that validly and reliably measures executive functions (80). The VST compromises three parts: (1) VST 1: naming the color of dots (red, blue, green, or yellow), (2) VST 2: naming the color of neutral words (e.g., words like “when” or “hardly” colored in red, blue, green, or yellow), and (3) VST 3: naming the color of “color words” printed in incongruent colors (e.g., word = red while word color = blue, etc.). In VST 3, interfering information is provided which requires interference control and response inhibition; the fast and automatic response of reading the words has to be inhibited and a more effortful color-naming response has to be produced. Each part contains 24 stimuli. Performance time [s] was recorded for each task and errors were counted.

##### Trail Making Test

The valid and reliable Trail Making Test (TMT) was used to assess psychomotor speed and executive functioning (81). In the first part of the test (TMT A), participants had to connect randomly allocated, encircled numbers from 1 to 25 in ascending order as fast as possible. In the second part of the test (TMT B), the stimuli comprise encircled letters and numbers. The randomly allocated numbers and letters have to be connected in ascending numerical and alphabetical order alternatingly as fast as possible (e.g., 1 – A – 2 – B – 3 – C – …). TMT A measures visual exploration skills and psychomotor processing speed whereas TMT B additionally assesses cognitive flexibility. In both parts, a short practice session was conducted. Time [s] for completing the tasks was recorded and errors were counted.

##### Wechsler Memory Scale-Revised (two subtests)

Further cognitive performance was evaluated with two subtests of the Wechsler Memory Scale-Revised (WMS-R) (82). The first subtest of the WMS-R, the digit span forward task (later on called WMS-R forward), assessed the short-term attention span and information processing speed. Participants had to remember and repeat digit sequences, which were read aloud and monotonously by the tester, in the correct order. The second subtest of the WMS-R, the digit span backward task (later on called WMS-R backward), was used to evaluate working memory capacity. Participants had to repeat the digit sequences in reversed order. Span length covered two to eight digits whereas for each length, two trials were presented to the participant prior to increasing sequence length. For every correct replication of a digit sequence, one point was scored, summing up to a total point score for each subtest. Moreover, the length of the longest correctly repeated digit sequence was recorded as the maximum span.

#### MRI (Image) Acquisition and Preprocessing

MRI measurements were conducted at cereneo (Neurorehabilitation Clinic cereneo AG, Vitznau, Switzerland) with a 3T Philips Ingenia MRI scanner (Philips Healthcare, Best, The Netherlands) equipped with a 32-channel head coil. The whole-brain structural images were obtained using a three-dimensional T1-weighted spoiled gradient echo pulse sequence (180 slices, TR: 20 ms, TE: 2.3 ms, flip angle: 20°, FOV: 220 mm × 220 mm × 135 mm, matrix size: 224 × 192, resulting in a voxel size of 0.98 mm × 1.15 mm × 1.5 mm).

For pre-processing, we used the VBM12 toolbox (Voxel-Based Morphometry; Structural Brain Mapping Group, Department of Psychiatry, University of Jean, Germany) of SPM12 (Statistical Parametric Mapping; The Wellcome Trust Centre for Neuroimaging, Institute of Neurology, University College London, London, United Kingdom) running under MATLAB R2017b (The Mathworks, Natick, Massachusetts, USA). First, all images were visually checked for artifacts, structural abnormalities, and pathologies by a physician. In case of brain abnormalities, participants were excluded. Then, the standard VBM routines and default parameters were applied (83). The pre-processing procedure implemented in VBM consisted of a tissue classification and segmentation into gray matter (GM), white matter (WM) and cerebrospinal fluid (CSF), followed by high-dimensional spatial DARTEL (Diffeomorphic Anatomical Registration Through Exponentiated Lie Algebra) normalization into MNI (Montreal Neurological Institute) space (84). The spatially normalized GM volumes were then smoothed with an isotropic Gaussian kernel of 6 mm (for further analysis of the cerebellum) respective 10 mm (for further analysis of the frontal cortex and the hippocampus) full width at half maximum (FWHM). The normalized and smoothed GM images were used for statistical analysis. Additionally, whole-brain GM, WM and CSF volumes in native space were calculated and summed to yield the total intra-cranial volume. Moreover, bilateral hippocampal volume (sum of right and left hippocampus volume) was calculated based on HV toolbox (Hippocampal Volumetry; jung diagnostics GmbH, Germany) (85) in SPM12.

### Statistical Analyses

#### Physical and Cognitive Data

The statistical analyses of the behavioral data were performed using SPSS 23.0 for Windows (SPSS Inc, Chicago, Illinois, USA), the R 3.5.2 software (The R Foundation for Statistical Computing, Vienna, Austria) and the package nparLD (86, 87). A *p*-value of *p* < 0.05 was considered significant. Per protocol analyses were performed. Adherence rate to the intervention was assessed with the training diary. For inclusion into analysis, a training adherence rate of 70% was predefined for the participants.

Descriptive statistics (median and interquartile range or frequencies) were reported for all data. The baseline group differences in the anthropometric data were tested using Mann Whitney U test or 2-dimensional (Person) Chi Square test. As common assumptions, in particular normality distribution of residuals and homogeneity of variance, were violated in most data, we used a rank-based non-parametric variance analysis (ANOVA-type statistic). The interaction effect of group (training, control) and time (pre, post) was investigated using the method described by Brunner and Langer (88) for non-parametric (rank-based) analysis of longitudinal data in factorial designs. Descriptions of how this approach works can be found elsewhere (88, 89). In case of a significant interaction effect, the Wilcoxon signed-rank test was used for *post hoc* analyses to investigate the within-group differences. For the ANOVA-type statistic, effect size was calculated by partial eta-square ($\eta_{\text{p}}^{2}$), expressing the amount of variance explained in the dependent variables by the respective effect (90). For the *post hoc* analyses, effect size r according to Cohen (91) was reported. Effect size r = 0.10 indicates a small effect, *r* = 0.30 a medium effect, and *r* ≥ 0.50 a large effect. For exploratory reasons, within-group pre- vs. post-comparisons and effect sizes *r* have been calculated for all the functional measures.

#### MRI Data

For statistical analyses of the MRI data, the VBM12 toolbox was used (Voxel-Based Morphometry; Structural Brain Mapping Group, Department of Psychiatry, University of Jean, Germany) of SPM12 (Statistical Parametric Mapping; The Wellcome Trust Centre for Neuroimaging, Institute of Neurology, University College London, London, United Kingdom) running under MATLAB R2017b (The Mathworks, Natick, Massachusetts, USA). Following the preprocessing of MRI images, standard parametric procedures were used, as the residuals are most likely to be normally distributed once the segmented images have been smoothed. A two-way repeated measures ANOVA with group (training, control) as between-subject factor and time (pre, post) as within-subject factor was calculated for the preprocessed GM images for predefined regions of interest (ROI). These ROIs were the hippocampus, frontal lobe, and cerebellum, for which masks were taken in MNI space from the wfu-pickatlas (92). Total intra-cranial volume, age and sex were included as covariates of no interest in all the analyses to correct for brain volume differences related to age and sex. All analyses were thresholded with a family-wise error (FWE)-corrected voxel significance level of *p* < 0.05. In addition, the repeated measures ANOVA was conducted for total brain GM volume as well as for hippocampal volume (in mm^3^).

To determine differences in change of GM volume between the training and control group after intervention, the interaction effect of time x group was reported. Furthermore, the main effect of time was reported to evaluate GM volume changes over time in both groups (age-related atrophy). Effect sizes were calculated by partial eta-square ($\eta_{\text{p}}^{2}$) (90).

All data generated and analyzed during this study are included in this published article.

## Results

**Figure 2:**
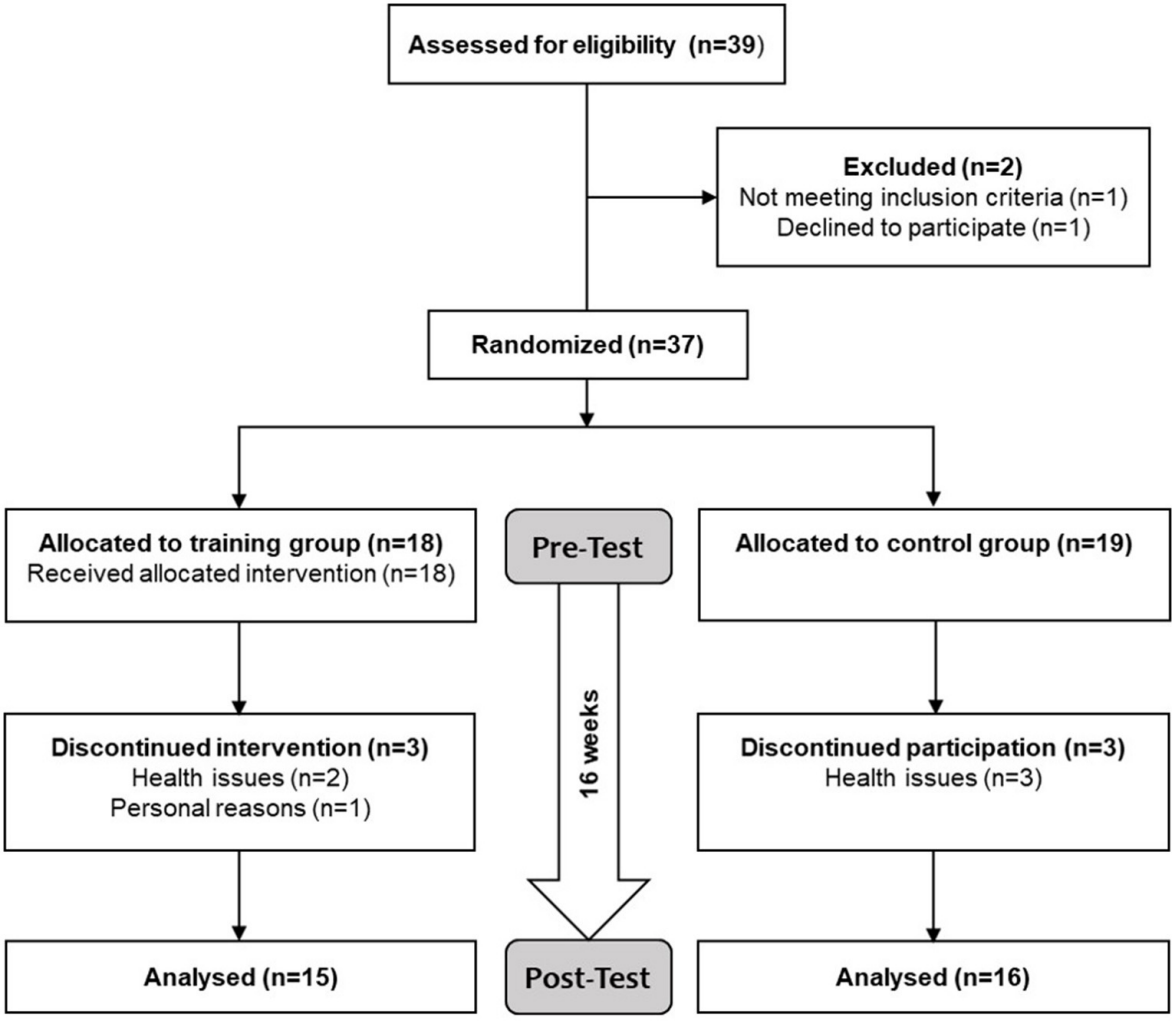
Study flow chart. Screening for eligibility included a health questionnaire and the Mini Mental Status Examination. Eligible participants were randomly assigned to either training or control group. The participants of the training group trained 3x/week à 30–40 min for 16 weeks while the participants of the control group continued with their normal daily living. Physical and cognitive functions as well as brain volume were assessed before and after the intervention period.

Out of the 37 older adults who were randomized (training, control), 31 participants completed the 16-week intervention period (mean age = 73.9 ± 6.4 years, range = 65–90 years, 16 female). Figure 2 shows the study flow chart. Since the six drop-outs were equally distributed to both groups and clear reasons for not completing the study were known, these participants were excluded from analysis. The drop-outs (mean age = 73.5 ± 8.0 years, range = 65–83 years, 5 female) were comparable to the remaining participants regarding their characteristics (*p* > 0.05). Table 1 summarizes baseline demographic data and screening values of participants who were included in the analysis. The two groups differed at baseline in age (*p* = 0.005), fear of falling (*p* = 0.013), and self-evaluation of balance (*p* = 0.049). All participants in the training group adhered to 70% or more of the total training sessions.

**Table 1:** Baseline demographic characteristics and screening values of analyzed participants.

**Participant characteristics**	**Training group (*n* = 15)**	**Control group (*n* = 16)**	**U or *X^**2**^*(df)**	***p***
Age [years]	77.0 ± 6.4 (65–90)	70.9 ± 5.0 (65–84)	49.5	0.005^*^
BMI [kg/m^2^]	24.2 ± 2.2 (20.9–28.1)	27.0 ± 5.1 (20.9–42.4)	77.0	0.089
Education [years]	13.3 ± 4.0 (8–25)	14.4 ± 2.6 (10–19)	79.0	0.101
MMSE Score	28.9 ± 1.1 (27–30)	29.2 ± 0.9 (27–30)	102.0	0.452
Female [*n*, %]	10 (66.7)	6 (37.5)	2.637(1)	0.104
**Fear of falling [*****n*****, %]**				
Never	7 (46.7)	14 (87.5)	8.755(2)	0.013^*^
Sometimes	8 (53.3)	1 (6.3)		
Often	0 (0.0)	1 (6.3)		
Always	0 (0.0)	0 (0.0)		
**Number of falls during last month**^+^ **[*****n*****, %]**				
Never	11 (73.3)	15 (93.8)	2.386(1)	0.122
Once	4 (26.7)	1 (6.3)		
More than once	0 (0.0)	0 (0.0)		
**Self-evaluation of health state [*****n*****, %]**				
Very good	1 (6.7)	2 (12.5)	3.972(3)	0.265
Good	12 (80.0)	11 (68.8)		
Medium	2 (13.3)	3 (18.8)		
Bad	0 (0.0)	0 (0.0)		
**Self-evaluation of balance [*****n*****, %]**				
Very good	1 (6.7)	2 (12.5)	7.849(3)	0.049^*^
Good	4 (26.7)	11 (68.8)		
Medium	8 (53.3)	3 (18.8)		
Bad	2 (13.3)	0 (0.0)		
**Self-evaluation of muscle strength [n, %]**				
Very good	1 (6.7)	2 (12.5)	4.540(3)	0.209
Good	5 (33.3)	9 (56.3)		
Medium	6 (40.0)	5 (31.3)		
Bad	3 (20.0)	0 (0.0)		
**Physical activity level [hours/week]**				
Intense physical activity	0 (0.0; 2.1)	0 (0.0; 0.4)	113.0	0.719
Moderate physical activity	2 (0.8; 5.0)^++^	3 (0.0; 7.5)	94.0	0.658

*Data are mean values ± standard deviation (range) or number of participants per category (absolute and relative frequency) as indicated*.

+ *Self-stated*.

++ *n = 13 due to missing values. Group differences were evaluated using Mann Whitney U test and 2-dimensional (Pearson) Chi Square test*.

* *p < 0.05, p-values are asymptotic sig. two-tailed. Bold values indicate significance. BMI, Body Mass Index; MMSE, Mini Mental State Examination*.

### Physical and Cognitive Functions

The descriptive statistics (median values and interquartile ranges) of all physical and cognitive measures are presented in Tables 2, 3, which include the results from the non-parametric variance analysis. For the dual-task gait analysis, data of one participant from the training group had to be excluded due to a violation of the testing protocol (prioritizing one task above the other). Due to technical issues, for two participants, data of one foot were used instead of averaging data of left and right foot.

**Table 2:** Interaction effects (group x time) in physical functions.

**Physical functions**	**Training group (*****n*** **= 15)**		**Control group (*****n*** **= 16)**		**Group x time interaction (*****n*** **= 31)**		
	**Pre**	**Post**	**Pre**	**Post**	***F*_**(1)**_**	***p***	**$\eta_{2}^{\text{p}}$**
**Gait analysis**							
**Speed mean [m/s]**							
ST walking DT walking DT costs in %	1.26 (1.12;1.37) 1.09 (0.95;1.25)^+^ 14.3 (8.6;15.8)^+^	1.29 (1.17;1.38) 1.13 (1.06;1.27)^+^ 10.1 (3.4;12.3)^+^	1.40 (1.24;1.48) 1.29 (1.14;1.38) 8.3 (5.5;11.1)	1.40 (1.31;1.46) 1.30 (1.22;1.36) 5.7 (4.8;8.4)	0.015 0.418^++^ 0.106^++^	0.904 0.518 0.745	<0.01 <0.01 <0.01
**Speed CV [%]**							
ST walking DT walking	7.0 (4.8;8.5) 6.8 (5.1;7.8)^+^	5.5 (4.9;6.0) 5.7 (4.8;6.5)^+^	5.3 (4.6;5.7) 5.8 (4.8;7.1)	4.8 (4.3;5.6) 5.6 (4.8;6.4)	1.299 1.018^++^	0.254 0.313	0.05 0.02
**Stride length mean [m]**							
ST walking DT walking DT costs in %	1.28 (1.22;1.40) 1.20 (1.13;1.34)^+^ 6.7 (5.2;9.0)^+^	1.31 (1.17;1.37) 1.24 (1.13;1.32)^+^ 4.1 (1.3;5.5)^+^	1.41 (1.35;1.46) 1.35 (1.27;1.41) 4.5 (3.1;5.7)	1.40 (1.35;1.46) 1.35 (1.32;1.40) 3.2 (1.4;5.6)	0.155 0.275^++^ 1.007^++^	0.693 0.600 0.316	<0.01 0.01 <0.01
**Stride length CV [%]**							
ST walking DT walking	4.5 (3.9;6.6) 4.5 (3.7;5.7)^+^	4.3 (3.8;4.7) 4.4 (3.8;5.1)^+^	3.8 (3.4;4.2) 4.1 (3.9;5.1)	3.8 (3.3;4.2) 4.0 (3.5;4.7)	0.359 0.057^++^	0.549 0.811	0.02 0.01
**Toe clearance mean [cm]**							
ST walking DT walking DT costs in %	1.94 (1.78;2.48) 1.98 (1.57;2.40)^+^ 7.6 (-2.6;14.4)^+^	2.06 (1.47;2.96) 2.14 (1.45;2.88)^+^ −2.3 (−8.2;8.6)^+^	1.92 (1.33;2.71) 1.72 (1.25;2.36) 0.4 (−10.5;7.8)	2.29 (1.84;2.94) 2.21 (1.62;3.03) 5.4 (−3.3;11.1)	0.617 0.716^++^ 2.538^++^	0.432 0.397 0.111	<0.01 <0.01 0.03
**Toe clearance CV [%]**							
ST walking DT walking	39.8 (29.2;46.9) 34.7 (31.1;51.6)^+^	35.2 (31.2;50.2) 36.3 (32.0;45.1)^+^	39.7 (26.0;61.4) 38.3 (25.7;48.5)	31.1 (22.8;44.9) 28.6 (21.0;48.7)	2.245 1.053^++^	0.134 0.305	0.02 <0.01
**Cycle duration mean [s]**							
ST walking DT walking DT costs in %	1.04 (1.01;1.07) 1.12 (1.09;1.17)^+^ −7.1 (−9.8;−3.6)^+^	1.04 (1.01;1.08) 1.09 (1.08;1.14)^+^ −4.9 (−7.4;−3.2)^+^	1.02 (1.00;1.09) 1.08 (1.05;1.14) −4.1 (−5.7;−1.7)	(0.99;1.08)1.05 (1.03;1.11) −3.7 (−4.7;−1.7)	0.034 0.630^++^ 0.195^++^	0.853 0.427 0.659	<0.01 0.02 0.03
**Cycle duration CV [%]**							
ST walking DT walking	3.3 (2.7;4.0) 3.7 (3.2;4.8)^+^	2.9 (2.5;3.2) 3.0 (2.7;3.7)^+^	2.6 (2.1;2.9) 2.9 (2.2;3.7)	2.3 (1.8;2.6) 3.0 (2.4;3.5)	0.032 2.456^++^	0.858 0.117	0.02 0.02
**Extended balance test of SPPB**							
Balance score	6 (5, 7)	6 (5, 6)	6.5 (6, 7)	6 (6, 7)	1.058	0.304	0.03
**Senior fitness test**							
30 s chair rises test 2 min stepping test	13 (12.0;16.5) 66 (59.5;82.0)	13 (13.0;15.5) 76 (69.5;83.5)	16.5 (14, 20) 74.5 (63, 89)	15.5 (12.75;18.75) 78.5 (73.75;81.50)	5.076 0.465	0.024^*^ 0.495	0.01 <0.01

*Data are median values (interquartile ranges). Non-parametric variance analysis was used to evaluate interaction effects (group × time) in physical functions*.

+ n = 14.

++ n = 30.

* *p < 0.05, bold values indicate significance. ST, single-task; DT, dual-task, DT costs are calculated as (ST–DT)/ST−100. SPPB, Short Physical Performance Battery; CV, coefficient of variation*.

**Table 3:** Interaction effects (group x time) in cognitive functions.

**Cognitive functions**	**Training group (*****n*** **= 15)**		**Control group (*****n*** **= 16)**		**Group x time interaction (*****n*** **= 31)**		
	**Pre**	**Post**	**Pre**	**Post**	***F*_**(1)**_**	***p***	**$\eta_{2}^{\text{p}}$**
**Trail Making Test (TMT)**							
**TMT A**							
Time [s] Errors	43 (35.5;53.5) 0 (0;0)	38 (33.5;41) 0 (0;0)	37.5 (32.75;45.75) 0 (0; 0)	33 (26.5;47.75) 0 (0; 0)	0.014 0.244	0.904 0.621	<0.01 <0.01
**TMT B**							
Time [s] Errors	123 (91;161.5) 0 (0;1)	104 (87;119.5) 1 (0;2)	83.5 (69.75;123.5) 0 (0; 1)	91 (71;108.25) 1 (0; 2)	1.508 0.151	0.219 0.698	0.01 <0.01
**Wechsler Memory Scale-Revised**							
Forward score Forward span Backward score Backward span	7 (6, 9) 6 (5, 7) 5 (4, 6) 4 (4, 4)	6 (6;7.5) 6 (5, 6) 6 (5;6.5) 4 (4, 7)	7 (5.75;8) 6 (5, 6) 6 (5.75;7.25) 5 (4, 5)	7 (6.75;8) 6 (6;6.25) 6 (5;7.25) 4 (4;5.25)	8.504 4.309 2.868 5.872	0.004^*^ 0.038^*^ 0.090 0.015^*^	0.04 0.03 0.02 0.02
**Victoria Stroop Test (VST)**							
**VST 1**							
Time [s] Errors	15 (13;16.5) 0 (0;1)	14 (13.5;16) 0 (0;1)	13.5 (12;16.25) 0 (0; 1)	13 (11.75;15.5) 0 (0;0)	0.002 2.174	0.963 0.140	<0.01 0.02
**VST 2**							
Time [s] Errors	20 (17.5;24) 0 (0;1)	20 (18, 22) 0 (0;1)	17.5 (14.75;21.25) 0 (0;0.25)	16 (13.75;21) 0 (0;1)	0.206 0.253	0.650 0.615	<0.01 <0.01
**VST 3**							
Time [s] Errors	36 (27.5;41.5) 1 (1;2.5)	30 (25.5;34) 1 (1, 2)	28.5 (23.75;38.25) 1 (0;2)	27.5 (22.25;35.25) 1 (0;2)	2.537 0.006	0.046^*^ 0.938	0.11 <0.01

*Data are median values (interquartile ranges). Non-parametric variance analysis was used to evaluate interaction effects (group x time) in physical functions*.

* *p < 0.05, bold values indicate significance*.

Non-parametric variance analysis showed no significant interaction effects for any of the physical function measures (*p* > 0.05) with the exception of an interaction effect in the 30 s chair rises test [*F*_(1)_ = 5.076, *p* = 0.024, ${\text{n}}_{\text{p}}^{2}$ = 0.01]. *Post hoc* analyses revealed no significant within-group changes comparing pre- and post-measurements.

Regarding cognitive functions, a significant interaction effect was present in the VST 3 [*F*_(1)_ = 2.537, *p* = 0.046, ${\text{n}}_{\text{p}}^{2}$ = 0.11]. *Post hoc* analyses revealed that the training group significantly improved in VST 3 after intervention (z = −3.051, *p* = 0.001, *r* = 0.56), whereas the control group did not (z = −1.885, *p* = 0.060, *r* = 0.33). Significant interaction effects were found in the WMS-R forward score [*F*_(1)_ = 8.504, *p* = 0.004, ${\text{n}}_{\text{p}}^{2}$ = 0.04] and WMS-R forward span [*F*_(1)_ = 4.309, *p* = 0.038, ${\text{n}}_{\text{p}}^{2}$ = 0.03]. Within-group pre-post comparisons showed that the control group significantly improved in WMS-R forward score (z = −2.268, *p* = 0.023, *r* = 0.40), whereas the training group did not (z = −1.409, *p* = 0.159, *r* = 0.26). A significant interaction effect was present in the WMS-R backward span [*F*_(1)_ = 5.872, *p* = 0.015, ${\text{n}}_{\text{p}}^{2}$ = 0.02]. *Post hoc* analyses revealed that the training group performed significantly better in WMS-R backward span after the intervention (z = −2.126, *p* = 0.033, *r* = 0.39), whereas the control group did not (z = −0.432, *p* = 0.666, *r* = 0.08). Further exploratory analyses (within-group pre- vs. post-comparisons for all functional measures) are shown in the Supplementary Material.

### Brain Volume

Eleven participants could not conduct MRI measurements or their MRI images could not be analyzed due to metal pieces in the body from surgeries (4 participants), claustrophobia (1 participant), intake of antidepressants (1 participant), loss of data (1 participant), rejection of MRI measurement due to personal reasons (2 participants) or dropping out from the study due to health-related issues (2 participants). No participant was excluded due to brain abnormalities or movement artifacts. MRI data of 12 participants of the training group and 14 participants of the control group were finally analyzed.

Repeated measures ANOVA did not reveal significant GM volume differences between the training and control group after intervention in the ROI analyses of the frontal cortex, the hippocampus and the cerebellum (significance level *p* < 0.05, FWE-corrected). Table 4 shows the significant main effects of time in frontal and hippocampal brain areas. Both groups showed a decrease in brain volume in these areas (right orbitofrontal cortex, right and left ventrolateral and ventromedial prefrontal cortex, left hippocampus) at the second measurement compared to the first.

**Table 4:** Main effects of time in structural MRI ROI analysis.

**Brain structure**	**BA**	**Hemisphere**	**Cluster size (# of voxels)**	**Peak Z**	**MNI coordinates**			***p* (FWE-corr)**
					**X**	**Y**	**Z**	
**Frontal cortex**								
Medial OFC	11	Right	3,440	6.596	8	54	−23	<0.001^*^
Ventrolateral PFC	46	Right	5,867	5.485	53	44	0	<0.001^*^
Ventrolateral PFC	10	Left	534	5.069	−33	49	−4	0.002^*^
Ventromedial PFC	–	Left	436	4.948	−14	54	−7	0.003^*^
Ventromedial PFC	10	Right	140	4.658	13	48	3	0.011^*^
**Hippocampus**	–	Left	332	4.340	−26	−6	−20	0.002^*^

*Brain regions that show a significant change in volume comparing pre vs. post-measurements in both groups (training, control). No signficant time × group interaction effect. Two-way repeated measures ANOVA was used. n = 26*.

* *p < 0.05 (FWE-corr), bold values indicate significance. BA, Brodmann Area; FWE-corr, family-wise error-corrected; OFC, orbitofrontal cortex; PFC, prefrontal cortex*.

For extracted GM volumes (total brain GM and hippocampal volume), further analyses did not show a significant time x group interaction or a significant main effect of time on the total brain GM volume or the hippocampal volume (see Table 5). For analysis of the hippocampal volume, one participant was considered as outlier with a value > +2 standard deviations and was replaced by the mean.

**Table 5:** Main effects of time and interaction effects (group x time) in total brain gray matter volume and hippocampal volume.

**Brain volume [mm^**3**^]**	**Pre**	**Post**	
**Training group (*****n*** **= 12)**			
Total brain GM volume	646,659 ± 70,692	627,868 ± 41,726	
Hippocampus volume	9,141 ± 583	9,044 ± 624	
**Control group (*****n*** **= 14)**			
Total brain GM volume	647,644 ± 60,546	647,497 ± 61,489	
Hippocampus volume	9,466 ± 1,014	9,523 ± 1,045	
**ANOVA results**	***F***_**(1, 24)**_	***p***	$\eta_{2}^{\text{p}}$
**Main effect time**			
Total brain GM volume	1.799	0.192	0.07
Hippocampus volume	0.090	0.767	<0.01
**Group × time interaction**			
Total brain GM volume	1.744	0.199	0.07
Hippocampus volume	2.577	0.122	0.10

Data are mean values ± standard deviations. Two-way repeated measures ANOVA was used to evaluate main effects of time and interaction effects (group × time) in total brain GM volume and hippocampal volume. n = 26. *p < 0.05, bold values indicate significance. GM, gray matter.

### Other Outcomes

The subjectively reported activity, derived from the biweekly phone calls, were averaged to hours/week (h/w) over the study period. Participants of the training group reported an average cognitive activity (e.g., playing chess, learning a language, using an app) of 2.8 ± 0.7 h/w and 1.5 ± 0.5 h/w of intense physical activity (e.g., running or playing tennis) and 4.4 ± 0.6 h/w of moderate physical activity (e.g., walking or gardening). Participants of the control group reported to be on average 3.3 ± 0.6 h/w cognitively active and an intense physical activity of 1.4 ± 0.4 h/w and a moderate physical activity of 6.3 ± 1.1 h/w. Mann Whitney U tests showed no significant group differences in all three categories (*p* > 0.05).

During the intervention period, none of the participants suffered any adverse events related to the study intervention, especially no falls or injuries during measurements or training. Moreover, no fear of falling during exergame training was reported. Eight participants reported to have experienced a “critical moment” where they almost fell (e.g., tripping, slipping, swaying). Most “critical moments” occurred during strength exercises in a lunge position or during whole body rotations. Dizziness during training was reported by three participants (related to body rotations or exhaustion). Slight pain during training was stated by five participants whereas in two cases, the pain was related to the IMU attachment to the ankles with a wrong-size slap band. The other three participants reported slight knee and/or back pain.

## Discussion

In this study, we investigated the impact of a newly developed home-based exergame training on physical functions, cognition and brain volume compared to usual care in healthy older adults. The training group completed a 4-month intervention with physical-cognitive training embedded in the exergame. Our results showed an improvement in higher-order cognitive functions (executive functions) after exergame training compared to non-training controls. No training-related improvements were evident in physical functions or brain volume. However, the analysis of brain volume showed a significant reduction of frontal and hippocampal brain volume over time in both groups. In the following, we will discuss these findings, put the results in the context of existing knowledge and draw consequences for future studies using similar approaches.

### Training Effects on Cognitive Functions

Our results showed significant time x group interactions in favor of the training group for two measures of higher-order cognitive functions; inhibition and working memory. Inhibition is related to the capability to inhibit irrelevant information and responses, sometimes called interference control, as interfering information might trigger responses, which have to be controlled (93). Working memory means to memorize/maintain and manipulate information (“work” on them), also referred to as updating (93). Both cognitive skills are part of the executive functions, which are mainly controlled by frontal brain areas. Cognitive aging is accompanied by a decline, among others, in executive functions whereas a link can be hypothesized to the age-related changes in the frontal cortex (3). Executive functions play an important role in guiding through everyday life including problem-solving and planning and controlling of actions (93). Moreover, they contribute to safe gait as a higher risk of falling was observed in subjects with cognitive impairments including executive functioning deficits (94–98). Thus, the improvement of executive functioning in this study might be an outcome of high relevance as strengthened executive functions could enhance daily life performance of older adults.

Several other studies including combined physical-cognitive training as exergaming indicated that this training approach can boost cognitive functions, particularly executive functions (99–103). Thus, our study results are in line with the evidence provided by recent reviews and meta-analyses, which reported significant improvements in executive functions after exergame training (52, 53, 104). This might be related to the cognitive stimulation more or less explicitly embedded in the game scenario of exergames. But also coordinative exercises such as dancing as well as aerobic and strength exercises have been shown to improve cognitive functions (12, 105–111). In the Active@Home exergame, several components were included with rather high cognitive demands (step-based cognitive training, dancing, Tai Chi-inspired strength exercises). All components could have contributed to the improved cognitive functions in varying proportions and/or in synergistic interactions.

One executive function – mental flexibility – showed no significant time x group interaction in this intervention study which is in line with the results of a previous exergame study (101). Mental flexibility is related to the capability to switch between different tasks and to process and manipulate multiple information (93). Three possible explanations for the missing improvement should be mentioned: (a) The cognitive training was not challenging enough to train this cognitive function. (b) The improvement could not be assessed by the applied cognitive test supporting the need to use several cognitive tests to assess a cognitive outcome of interest as a latent construct behind the different tests (12). (c) Nevertheless, in further exploratory analyses comparing pre- vs. post-measurements within groups, the training group showed significant improvement in mental flexibility after the intervention, the control group did not. Thus, another explanation could be that the training effect was just not strong enough to get evident in a significant interaction effect.

A further, however rather surprisingly finding was the significant time x group interaction in the short-term attentional span in favor of the control group. As the control group was a passive control group and (physical and cognitive) activities beside of the activity due to the exergame intervention (in the training group) were assessed and comparable in both groups, activity-related improvements are likely to be excluded. Kent recently reviewed the WMS-R, of which the subtests to assess attentional span (WMS-R digit span forward) and working memory (WMS-R digit span backward) have been applied (112). Concerns have been expressed regarding the reliability and validity of this test battery, even though it is clinically used for over 75 years. Thus, caution might be required regarding the results measured with these subtests.

### Training Effects on Physical Functions

Another rather surprising result of this study was that no significant improvements got evident for any of the measured physical functions (gait parameters, muscle strength, balance and endurance). Previous studies have reported improvements in several physical functions of older adults after exergame training (e.g., in cardiovascular or musculoskeletal system) depending on the physical components and exercises that are integrated in the training (e.g., stepping or cycling) (54–56, 113). In exergame studies with similar interventions than in ours, gait parameters as walking speed have been shown to be enhanced after exergame training, especially under dual-task walking (103, 114, 115). Exergaming is discussed to train dual-task abilities, which are important in daily life activities and for safe gait and fall prevention in older adults (116, 117). Even though no significant interaction effects were evident in this study for gait parameters, in further exploratory analyses of pre- vs. post-comparisons within groups, the training group showed significant improvements in several gait parameters (walking speed, stride length and cycle duration) under dual-task walking in line with previous research. One might speculate that the effects have not been strong enough to get evident in the results of the variance analysis. However, the control group individuals showed a baseline walking speed around 1.4 m/s and, thus, seem to be “high performers” compared to reference values of walking speed for older adults, meaning there is little room for improvement (118). The gait improvements within the training group might be related to the improved executive functioning together with their lower baseline values.

Even though the physical exercises incorporated in the Active@Home exergame are supposed to target balance and strength, no improvements were evident in these functions. For example, dancing has been shown to positively influence coordinative skills and balance, whereas strength training with exercises similar to the ones incorporated in our intervention has been demonstrated to be effective in older adults (119–122). Also after step-based games, requiring the execution of rapid and well-directed steps, improvements in balance have been reported (123, 124). Moreover, in a previous pilot study of the Active@Home exergame (59), heart rate measures during training showed moderate physical intensity (an average heart rate of about 60% of the estimated maximal heart rate of the elderly participants), which is recommended to improve cardiovascular fitness (68). Nevertheless, no enhancement in aerobic endurance was evident in this study. Three aspects should be discussed related to the missing improvements in endurance, strength and balance.

One assumption is that insensitive and unsuitable assessments have been used. Some of the applied tests were originally designed for moderate-performing older adults or for clinical setting. Thus, they might have not been able to assess a sufficient range of performance in healthy (high-performing) older adults and to detect subtle differences (e.g., due to ceiling effects). For example, the SPPB was developed to assess older adults with low functionality and to evaluate their risk of disability (75). The SFT was developed for early detection of decline in physical functions (78, 125). Moreover, to assess strength and endurance, functional measures (rising from a chair, stepping) were used. Other measurement methods might have been much more accurate (e.g., leg press or maximal oxygen consumption). Another assumption relates to the duration of the intervention. Even though other exergame studies showed improvements in physical functions after several weeks of training and a very comparable training amount (e.g., min/week), prolonged intervention period might have uncover (greater) training effects (113). A last assumption may relate to the training load. An individually adapted, optimal training load has been discussed to be crucial to maximize training benefits and improve motor skills and physical fitness (72, 126, 127). Moreover, due to training-related improvements in the course of training, the training load has to be continuously adapted. This continuous adaptation leads to a progressively increasing training load. Participants in this study have been instructed that the current training load respective the chosen difficulty level should always provide an optimal challenge avoiding under- or overload. The exergame included various difficulty levels of exercises arranged in a progressive order. However, the training load and its progressive adaptation were not monitored or supervised and based on subjective estimates of the participants rather than on objective measurements (e.g., percentage of the individual maximal heart rate or the individual one repetition maximum). We hypothesize that some participants may have trained under their optimal load due to difficulties with self-perception and -evaluation. Additionally, although the Active@Home exergame training should provide moderate intense physical exercises based on the heart rate measures during training in a pilot study (59), it might be suggested, that higher intensity levels would have been required for physical adaptations.

### Structural Plasticity Effects

The study results revealed no increase in brain volume after exergame training. However, previous animal and human studies showed structural brain alternations after a variety of physical exercises in gray and white matter; for example after aerobic training (13, 24, 128, 129), after strength training (15), after coordinative exercises (130–134), after dancing (111, 121, 135) or after Tai Chi (136). Moreover, changes in brain structure have been reported after cognitive training (29–33, 137). To the best of our knowledge, only two studies have been conducted assessing the effects of exergame training on brain structure of older adults (57, 58). Ji and colleagues showed an increase in brain volume in fontal areas after Nintendo Wii training (57). Anderson-Hanley and colleagues reported similar findings after training with a specifically developed exergame (stationary bicycles with an interactive virtual component) (58). They also showed increased BDNF levels after exergame training. As exergame training combines physical exercise with cognitive stimulation, synergistic effects on neuroplasticity are assumed. Even though physical and cognitive training both seem to positively influence the brain, the underlying mechanisms might be different (36, 138). Indeed, physical activity has been shown to trigger cell proliferation of precursor cells and thereby inducing neurogenesis whereas cognitive stimulation seem to promote the survival and integration of the newborn cells into the functional networks (46, 47, 139). Based on these findings, the “facilitation-guidance-model” was proposed (46, 47): Physical exercise is assumed to facilitate neuroplastic processes while cognitive stimulation might guide the plastic changes. Although the Active@Home exergame incorporated combined physical-cognitive training, no positive influence on brain volume was evident after the 4-month intervention. Three potential reasons should be discussed.

First, most of the studies showing improvements in brain structure included longer intervention periods (6–12 months) (13, 15, 58, 111, 121, 133, 134). Hippocampal volume seems to be sensitive to the intervention duration: In a sample of older adults, an almost linear development in hippocampal volume enhancement was demonstrated after 6 and 12 months of aerobic training (13). In cognitive training studies, however intervention durations were around several weeks (29–33, 137). Thus, one might speculate that a longer training period with the Active@Home exergame could have led to effects on older adults' brain volume. Second, effects on the brain might have been evident, but they could probably not be measured by the applied measurement method. Other neuronal outcome measures might have been included (e.g., serum BDNF or IGF-1, diffusion tension imaging for white matter integrity and functional MRI). Third, the missing improvement in brain volume may relate to the training intensity. In general, moderate physical intensity is recommended for health benefits in older adults (corresponding to a heart rate of about 60–80% of the maximal heart rate) (68). An animal study by Ferris and colleagues showed that BDNF increase might be exercise intensity-dependent with higher intensity leading to higher expression (140). BDNF is known to trigger neuroplastic effects as synaptogenesis and neurogenesis (18, 141). Thus, it can speculated that higher physical training intensity would have shown (larger) positive effects. However, Ruscheweyh and colleagues compared outcomes of older adults engaged either in a medium- or a low-intense physical exercise to a no training control group (142). They found beneficial effects of physical activity independent of the intensity on cognitive functions and on frontal brain structure. Another study by Lövden and colleagues showed a positive effect on hippocampal volume after an exergame training with a very low physical intensity (slow walking) (143). Thus, the relationship between physical intensity and brain changes remains unclear. Nevertheless, indisputable is the fact that intense (metabolically demanding) exercises trigger adaptations in the cardiovascular and respiratory system, the metabolism and the immune system, and that, in turn, might have indirect effects on brain metabolism and processes (144, 145).

Moreover, this study revealed a significant time effect on brain volume. The ROI-analysis showed a reduction of frontal and hippocampal brain volume over time in both groups which has been observed in other interventional studies (32). This decrease in brain volume is consistent with numerous cross-sectional and longitudinal studies of aging (3, 146, 147). For example, Fjell and Waldhove reported an annual reduction of brain volume between 0.5 and 1.0% in most brain areas in older adults (3). Thus, this study result points to the brain degradation that seems to occur during normal aging.

### Limitations

One of the limitations of this study was that only a passive rather than an active control group was included. Nevertheless, social contact was kept stable between the two groups as exergame training was conducted at participants' homes (no supervised training sessions) and participants of both groups were regularly called to assess activities and safety issues. Another study limitation might be related to the choice of outcome measurements. On one hand, more than one test could have been used to assess an outcome of interest, thereby evaluating an underlying latent construct and increasing validity (12). On the other hand, no measures of daily life functioning have been included (28), meaning that transfer effects to daily life activities, which are tremendously important, have not been assessed. Moreover, some applied measurement methods have been originally developed for clinical setting, thus limiting the sensitive assessment of high performance. Regarding neuronal outcome measures, other outcomes than gray matter brain volume (e.g., white matter microstructure, BDNF and IGF-1) could have been considered. Additionally, relationship (potential correlation) between brain alterations and changes in performance should be assessed in future studies to highlight the meaningfulness of neuronal changes. Considering the rather fit participants in the study sample, ceiling effects in performance might have restricted informative value. Moreover, attention should be paid regarding generalization of the study results to overall older adults, again due to the rather fit and high-performing participants in this study. Another limitation of this study might be related to the rather small sample size, which might have limited the detection of significant training effects. Finally, during the exergame training, individual and progressive adaptation of training load was neither monitored nor based on objective measures. Further studies, especially with technology-based interventions, should consider automatic load adaptation.

## Conclusion

To prevent age-related decline and maintain brain health, continuous active interaction with environments that are demanding to sensory, motor and cognitive systems is necessary. Training-induced plasticity is evident on behavioral as well as on neuronal level also in higher age. Exergame training seems to be a motivating and promising option for simultaneous physical-cognitive training in older adults. Our study showed an improvement in higher-order cognitive functions (executive functions) after exergame training compared to the passive control group. While comparing our study results to the findings of other exergame studies, it got evident that this comparison is limited due to a large heterogeneity in exergame characteristics. Even though physical exercise is by definition a part of exergame training, the physical exercise component of an exergame might be very different in various exergames. One exergame training might be based on walking, running or cycling, another exergame training might require balance-shifts, stepping or jumping. Thereby, physical activity embedded in an exergame can be more or less intense respective metabolically demanding. The same heterogeneity can be found for cognitive stimulation incorporated in exergame training. Exergaming might require very basal but also rather specific and complex cognitive functions and processes. To summarize, a huge variety exists in physical and cognitive demands of exergame training. Therefore, benefits of exergame training might vary depending on the exergame characteristics. Especially regarding neuronal outcomes, different mechanisms might be triggered by exergames with various metabolic and cognitive demands. Further research is needed to assess the training conditions and requirements of exergame training to optimally enhance performance and brain structure. We suggest that regular physical-cognitive training with an individually adapted optimal training challenge might lead to both, greater improvements in cognitive and physical functions as well as measurable changes in brain structure.

## Data Availability Statement

All datasets generated for this study are included in the article/Supplementary Material.

## Ethics Statement

The studies involving human participants were reviewed and approved by Ethics committee of Nordwest- und Zentralschweiz (EKNZ, 2018-00510). The participants provided their written informed consent to participate in this study.

## Author Contributions

MA, MF, and JP developed the research question under the lead of EB. The concept and design were established by MA, MF, and JP, while AS, EB, and AL acted as methodological council. MA, MF, and JP conducted data acquisition, analysis, and interpretation of the results with edition and improvements by AS, EB, and KL. KL supported the MRI data analysis and interpretation. LZ was the study physician checking all MRI images for structural abnormalities and pathologies. VG was mainly responsible for exergame development including algorithms for movement evaluation. During the study intervention, VG provided technical support. MA produced the first version of the manuscript. AS and EB substantially revised the manuscript to bring it to its current version. All authors have read and approved the final manuscript.

### Conflict of Interest

The authors declare that the research was conducted in the absence of any commercial or financial relationships that could be construed as a potential conflict of interest.
