# Supplementary material for: Effects of an In-home Multicomponent Exergame Training on Physical Functions, Cognition, and Brain Volume of Older Adults: A Randomized Controlled Trial
Source: Front Med (Lausanne). 2020 Jan 28;6:321. doi: 10.3389/fmed.2019.00321 (PMC6997483; doi:10.3389/fmed.2019.00321)
Supplement: Supplementary file 1 [file Table_1.docx]

Supplementary Table 1 | Within-group pre- vs. post-comparisons for physical functions.

| **Training group (n = 15)** | **Pre** | **Post** | **z** | **p** | **r** |
| --- | --- | --- | --- | --- | --- |
| **Gait analysis** | | | | | |
| **Speed Mean [m/s]**  ST walking  DT walking^+^  DT costs in %^+^ | 1.26 (1.12;1.37)  1.09 (0.95;1.25)  14.3 (8.6;15.8) | 1.29 (1.17;1.38)  1.13 (1.06;1.27)  10.1 (3.4;12.3) | -1.477  -2.794  -2.229 | .151  **.003***  **.025*** | .27  .53  .42 |
| **Speed CV [%]**  ST walking  DT walking^+^ | 7.0 (4.8;8.5)  6.8 (5.1;7.8) | 5.5 (4.9;6.0)  5.7 (4.8;6.5) | -2.215  -2.291 | **.026***  **.020*** | .40  .43 |
| **Stride length Mean [m]**  ST walking  DT walking^+^  DT costs in %^+^ | 1.28 (1.22;1.40)  1.20 (1.13;1.34)  6.7 (5.2;9.0) | 1.31 (1.17;1.37)  1.24 (1.13;1.32)  4.1 (1.3;5.5) | -1.022  -2.040  -2.103 | .330  **.042***  **.035*** | .19  .39  .40 |
| **Stride length CV [%]**  ST walking  DT walking^+^ | 4.5 (3.9;6.6)  4.5 (3.7;5.7) | 4.3 (3.8;4.7)  4.4 (3.8;5.1) | -1.363  -0.973 | .188  .358 | .25  .18 |
| **Toe clearance Mean [cm]**  ST walking  DT walking^+^  DT costs in %^+^ | 1.94 (1.78;2.48)  1.98 (1.57;2.40)  7.6 (-2.6;14.4) | 2.06 (1.47;2.96)  2.14 (1.45;2.88)  -2.3 (-8.2;8.6) | -0.511  -1.224  -1.287 | .639  .241  .217 | .09  .23  .24 |
| **Toe clearance CV [%]**  ST walking  DT walking^+^ | 39.8 (29.2;46.9)  34.7 (31.1;51.6) | 35.2 (31.2;50.2)  36.3 (32.0;45.1) | -0.284  -0.220 | .804  .855 | .05  .04 |
| **Cycle duration Mean [s]**  ST walking  DT walking^+^  DT costs in %^+^ | 1.04 (1.01;1.07)  1.12 (1.09;1.17)  -7.1 (-9.8;-3.6) | 1.04 (1.01;1.08)  1.09 (1.08;1.14)  -4.9 (-7.4;-3.2) | -0.568  -2.166  -1.915 | .599  **.030***  .058 | .10  .41  .36 |
| **Cycle duration CV [%]**  ST walking  DT walking^+^ | 3.3 (2.7;4.0)  3.7 (3.2;4.8) | 2.9 (2.5;3.2)  3.0 (2.7;3.7) | -1.704  -3.045 | .095  **.001*** | .31  .58 |
| **Extended Balance Test of SPPB** | | | | | |
| Balance score | 6 (5;7) | 6 (5;6) | -1.140 | .250 | .21 |
| **Senior Fitness Test** | | | | | |
| 30sec chair rises test  2min stepping test | 13 (12.0;16.5)  66 (59.5;82.0) | 13 (13.0;15.5)  76 (69.5;83.5) | -0.942  -1.506 | .396  .139 | .17  .27 |
| **Control group (n = 16)** | **Pre** | **Post** | **z** | **p** | **r** |
| **Gait analysis** |  |  |  |  |  |
| **Speed Mean [m/s]**  ST walking  DT walking  DT costs in % | 1.40 (1.24;1.48)  1.29 (1.14;1.38)  8.3 (5.5;11.1) | 1.40 (1.31;1.46)  1.30 (1.22;1.36)  5.7 (4.8;8.4) | -0.776  -1.655  -1.293 | .464  .105  .211 | .14  .29  .23 |
| **Speed CV [%]**  ST walking  DT walking | 5.3 (4.6;5.7)  5.8 (4.8;7.1) | 4.8 (4.3;5.6)  5.6 (4.8;6.4) | -0.724  -0.052 | .495  .980 | .13  .01 |
| **Stride length Mean [m]**  ST walking  DT walking  DT costs in % | 1.41 (1.35;1.46)  1.35 (1.27;1.41)  4.5 (3.1;5.7) | 1.40 (1.35;1.46)  1.35 (1.32;1.40)  3.2 (1.4;5.6) | -1.810  -1.603  -1.396 | .074  .117  .175 | .32  .28  .25 |
| **Stride length CV [%]**  ST walking  DT walking | 3.8 (3.4;4.2)  4.1 (3.9;5.1) | 3.8 (3.3;4.2)  4.0 (3.5;4.7) | -0.465  -0.569 | .669  .597 | .08  .10 |
| **Toe clearance Mean [cm]**  ST walking  DT walking  DT costs in % | 1.92 (1.33;2.71)  1.72 (1.25;2.36)  0.4 (-10.5;7.8) | 2.29 (1.84;2.94)  2.21 (1.62;3.03)  5.4 (-3.3;11.1) | -1.655  -1.706  -0.362 | .105  .093  .744 | .29  .30  .06 |
| **Toe clearance CV [%]**  ST walking  DT walking | 39.7 (26.0;61.4)  38.3 (25.7;48.5) | 31.1 (22.8;44.9)  28.6 (21.0;48.7) | -1.965  -1.551 | .051  .130 | .35  .27 |
| **Cycle duration Mean [s]**  ST walking  DT walking  DT costs in % | 1.02 (1.00;1.09)  1.08 (1.05;1.14)  -4.1 (-5.7;-1.7) | 1.01 (0.99;1.08)  1.05 (1.03;1.11)  -3.7 (-4.7;-1.7) | -0.310  -0.776  -0.569 | .782  .464  .597 | .05  .14  .10 |
| **Cycle duration CV [%]**  ST walking  DT walking | 2.6 (2.1;2.9)  2.9 (2.2;3.7) | 2.3 (1.8;2.6)  3.0 (2.4;3.5) | -0.931  0.000 | .375  1.000 | .16  <.01 |
| **Extended Balance Test of SPPB** | | | | | |
| Balance score | 6.5 (6;7) | 6 (6;7) | -1.341 | .182 | .24 |
| **Senior Fitness Test** |  |  |  |  |  |
| 30sec chair rises test  2min stepping test | 16.5 (14;20)  74.5 (63;89) | 15.5 (12.75;18.75)  78.5 (73.75;81.50) | -1.256  -0.026 | .242  .990 | .22  <.01 |

Data are median values (interquartile ranges). Wilcoxon signed-rank test was used. ^+^n = 14. *p < .05, p-values are exact sig. two-tailed, bold values indicate significance. Effect size r = .10 indicates a small effect, r = .30 a medium effect, and r ≥ .50 a large effect. Abbreviations: ST = single-task. DT = dual-task. DT costs are calculated as (ST – DT)/ST x 100. SPPB = Short Physical Performance Battery. CV = coefficient of variation.

Supplementary Table 2 | Within-group pre- vs. post-comparisons for cognitive functions.

| **Training group (n = 15)** | **Pre** | **Post** | **z** | **p** | **r** |
| --- | --- | --- | --- | --- | --- |
| **Trail Making Test (TMT)** | | | | | |
| **TMT A**  Time [s]  Errors | 43 (35.5;53.5)  0 (0;0) | 38 (33.5;41)  0 (0;0) | -1.602  -1.414 | .115  .500 | .29  .26 |
| **TMT B**  Time [s]  Errors | 123 (91;161.5)  0 (0;1) | 104 (87;119.5)  1 (0;2) | -2.166  -1.674 | **.030***  .125 | .40  .31 |
| **Wechsler-Memory Scale-Revised** | | | | | |
| Forward score  Forward span  Backward score  Backward span | 7 (6;9)  6 (5;7)  5 (4;6)  4 (4;4) | 6 (6;7.5)  6 (5;6)  6 (5;6.5)  4 (4;5) | -1.409  -1.155  -1.767  -2.126 | .159  .248  .077  **.033*** | .26  .21  .32  .39 |
| **Victoria Stroop Test (VST)** | | | | | |
| **VST 1**  Time [s]  Errors | 15 (13;16.5)  0 (0;1) | 14 (13.5;16)  0 (0;1) | -0.885  0.000 | .427  1.000 | .16  <.01 |
| **VST 2**  Time [s]  Errors | 20 (17.5;24)  0 (0;1) | 20 (18;22)  0 (0;1) | -1.843  -0.577 | .061  .781 | .33  .10 |
| **VST 3**  Time [s]  Errors | 36 (27.5;41.5)  1 (1;2.5) | 30 (25.5;34)  1 (1;2) | -3.051  -0.060 | **.001***  .992 | .56  .01 |
| **Control group (n = 16)** | **Pre** | **Post** | **z** | **p** | **r** |
| **Trail Making Test (TMT)** |  |  |  |  |  |
| **TMT A**  Time [s]  Errors | 37.5 (32.75;45.75)  0 (0; 0) | 33 (26.5;47.75)  0 (0; 0) | -1.450  -0.577 | .155  1.000 | .26  .11 |
| **TMT B**  Time [s]  Errors | 83.5 (69.75;123.5)  0 (0; 1) | 91 (71;108.25)  1 (0; 2) | -0.543  -2.041 | .605  .063 | .10  .37 |
| **Wechsler-Memory Scale-Revised** | | | | | |
| Forward score  Forward span  Backward score  Backward span | 7 (5.75;8)  6 (5;6)  6 (5.75;7.25)  5 (4;5) | 7 (6.75;8)  6 (6;6.25)  6 (5;7.25)  4 (4;5.25) | -2.268  -1.890  -0.811  -0.432 | **.023***  .059  .417  .666 | .40  .33  .14  .08 |
| **Victoria Stroop Test (VST)** | | | | | |
| **VST 1**  Time [s]  Errors | 13.5 (12;16.25)  0 (0; 1) | 13 (11.75;15.5)  0 (0;0) | -0.472  -2.236 | .689  .063 | .08  .40 |
| **VST 2**  Time [s]  Errors | 17.5 (14.75;21.25)  0 (0;0.25) | 16 (13.75;21)  0 (0;1) | -2.582  -0.816 | **.010***  .750 | .46  .14 |
| **VST 3**  Time [s]  Errors | 28.5 (23.75;38.25)  1 (0;2) | 27.5 (22.25;35.25)  1 (0;2) | -1.885  -0.036 | .060  1.000 | .33  .01 |

Data are median values (interquartile ranges). Wilcoxon signed-rank test was used. *p < .05, p-values are exact sig. two-tailed, bold values indicate significance. Effect size r = .10 indicates a small effect, r = .30 a medium effect, and r ≥ .50 a large effect.
